# Prevalence and Correlates of Anxiety and Depressive Symptoms in Patients With and Without Multi-Drug Resistant Pulmonary Tuberculosis in China

**DOI:** 10.3389/fpsyt.2021.674891

**Published:** 2021-09-07

**Authors:** Kewei Liu, Yidan Zhang, Shilin Qu, Wenying Yang, Liyuan Guo, Liqun Zhang

**Affiliations:** ^1^Department of Tuberculosis, Capital Medical University, Beijing Chest Hospital, Beijing, China; ^2^Chinese Academy of Sciences Key Laboratory of Mental Health, Institute of Psychology, Chinese Academy of Sciences, Beijing, China; ^3^Department of Psychology, University of Chinese Academy of Sciences, Beijing, China; ^4^Department of Multidrug Resistance Tuberculosis, Jingzhou Infectious Diseases and Chest Hospital, Jingzhou, China

**Keywords:** tuberculosis, TB stigma, social supports, depression, anxiety, drug-resistant

## Abstract

China is still among the 30 high-burden tuberculosis (TB) countries in the world and TB remains a public health concern. TB can be a cause of mental illness, with prolonged treatment and several anti-TB drugs leading to extreme mental health problems such as depression and anxiety in TB patients. To investigate the prevalence of anxiety and depressive symptoms among TB patients, and to explore whether drug resistance is a covariate for depressive and anxiety symptoms, a total of 167 pulmonary tuberculosis patients were enrolled in this study, which was conducted from January 1 to September 30, 2020. Data were collected, using a structured questionnaire with a demographic component, the Hospital Anxiety and Depression Scale (HADS), General Health Questionnaire 20 (GHQ-20), the Tuberculosis-related Stigma Scale (TSS) and the Social Support Rating Scale (SSRS). Association between demographics, disease/treatment characteristics, stigma, social support, and anxiety/depression symptoms were investigated either based on Pearson's correlation coefficient or group comparisons based on independent *t*-test (or Mann-Whitney *U-*test) Multiple linear stepwise regression analysis was used for determining the predictors of anxiety and depression. The results showed that multi-drug resistance pulmonary tuberculosis patients were associated with anxiety challenges. Multiple linear regression analysis indicated that self-esteem accounted for 33.5 and 38% of the variation in anxiety and depression, respectively. This shows that among tuberculosis patients, self-esteem is the factor that could most explains the depression and anxiety symptoms of patients, suggesting that we may could through improving the environment, society, and family respect and tolerance of tuberculosis patients, thereby improving the mental health of tuberculosis patients.

## Introduction

Tuberculosis (TB) is a chronic infectious disease caused by mycobacterium tuberculosis. It is one of the leading causes of morbidity and mortality worldwide (1). According to the World Health Organization's 2019 report, there are about one-quarter of the world's population has latent TB,10 million people are infected with TB each year, 484,000 of whom are drug-resistant TB (of which 78% have the MDR-TB), and 1.5 million people die of TB each year. China is still among the 30 high-burden TB countries in the world, and TB remains a public health concern (2).

Anxiety and depression are the most common mental disorders in the general population. The presence of anxiety and depression has a negative impact on quality of life, healthcare costs and self-care (3). Patients with TB may suffer from mental disorders as a result of long-term treatment, anti-TB drug side effects and TB relapses (4). Studies on the prevalence of anxiety and depression in TB patients in different countries or regions showed that the prevalence of anxiety and depression in TB patients is about 40.67–72.88% and 9.93–61%, respectively (3, 5–8). A study in China showed that the prevalence of anxiety and depression in TB patients is about 18.37 and 18.13%, respectively (4).

TB can be a cause of mental illness, with prolonged treatment and several anti-TB drugs (such as cycloserine) leading to extreme mental health problems such as anxiety and depression (4). Mental illness complications have been associated with antituberculosis therapy since the 1950s. The possible environmental and genetic factors of anti-TB medication-induced adverse reactions have always been the matter of concern (9). In addition, TB patients continue to experience significant social discrimination and increased stigma (10). Since they have tuberculosis, the patients might be discriminated at workplace and public areas as the disease is airborne. In addition, prevailing weakness caused due to the disease would lead to inefficient work and result in unemployment and negative events in life. There is evidence that these factors can cause patients to stop treatment, increase feelings of alienation, and lead to extreme forms of self-harm, including suicide (11–13). However, little is known about the prevalence of anxiety and depression among TB patients in China, studies on effect of MDR-TB/ treatment in causing anxiety and depression are meager.

This study aimed to investigate the prevalence and correlates of anxiety and depressive symptoms among pulmonary TB patients, and explore whether TB-drug resistance was associated with greater anxiety and depressive symptoms.

## Materials and Methods

### Study Design

A cross-sectional design was conducted.

### Study Site

This study was conducted in two hospitals: (1) the Beijing Chest Hospital, Beijing China and (2) JingZhou Chest Hospital, JingZhou city, HuBei province, that care for patients with TB.

### Subjects

From January 1 to September 30, 2020, a convenience sample of 167 patients who were hospitalized, under treatment, or newly diagnosed were recruited. Inclusion criteria: (1) TB patients meeting the diagnostic criteria: drug-sensitive TB was based on drug susceptibility testing which showed that mycobacterium tuberculosis were susceptible to all of the anti-TB drugs; MDR-TB was based on drug susceptibility testing which showed that mycobacterium tuberculosis were resistant to at least isoniazid and rifampicin simultaneously. (2) No other serious somatic diseases; (3) Clear consciousness and cognitive abilities; (4) Ability to complete the questionnaire independently. Exclusion criteria: (1) Pregnant, children, seriously ill or with communication problems; (2) Patients with serious diseases, such as cancer, HIV; (3) The diagnosis of TB is not clear; (4) Antidepressant use during the investigation period.

### Data Collection

Data were collected using an online structured questionnaire and filled out by the patients using their own smartphones or tablets. The questionnaire included demographic information such as gender, age, education, course of TB, drug resistance states, times of treatments, treatment outcome, etc. The Hospital Anxiety and Depression Scale (HADS) and The General Health Questionnaire 20 (GHQ-20) were included in the questionnaire to evaluate patient depressive and anxiety symptoms. The Tuberculosis-related Stigma Scale (TSS) was used to evaluate stigma of the patients. The Social Support Rating Scale (SSRS) was used to evaluate the social support. All the scales were the Chinese versions, which were valid and efficient.

#### The Hospital Anxiety and Depression Scale

The scale is a 14-items self-rating scale, addressing anxiety/depression related issues in the previous 2 weeks. The Chinese version of HADS is wildly used in china and was found to be a reliable measurement tool for excluding depression and anxiety in patients with the Cronbach's alpha coefficients of the HADS-A and HADS-D subscales were 0.753 and 0.764, respectively (14). It is composed of two subscales, anxiety (HADS-A) and depression (HADS-D), each consisting of 7 items. Likert 4 score (0–3 points) is used for each item. Zigmond divided the subscale scores as follows: 0–7 in normal state, 8–10 in suspicious state, and 11–21 in positive state (15). Therefore, in this study, the critical value is 8.The overall HADS score was divided into normal state (0–14) and mental illness symptom (15–42). HADS has been widely used in the screening of clinical patients' psychological problem. A previous study reported that the correlation coefficient of the two HADS subscales was 0.40–0.74 with an average of 0.56, and Cronbach's coefficient was 0.83 and 0.82, respectively, which proved that the scale had good reliability and validity (16).

#### General Health Questionnaire 20

The General Health Questionnaire was revised in Chinese version (17). This self-report questionnaire comprises a total of 20 items, including self-esteem (9 items), depression scale (6 items), and anxiety scale (5 items), participants were all asked to use “yes” or “no” to indicate how they had felt in last 2 weeks. From 1 to 9, self-esteem scale is a measure of the positive direction of mental health. The score range is 0–9. Questions 10–15 are the depression scale, and questions 16–20 are the anxiety scale. These two scales measure the negative direction of mental health.

#### The Tuberculosis-Related Stigma Scale

The TSS was a self-rating scale which developed by Yang Tingting to assess patients' perceptions of TB stigma in the last 2 weeks, which has 9 items. The score of all items is the patient's stigma score, which ranges from 0 to 27 points. The higher score refers to the higher stigma of tuberculosis patient. The overall Cronbach coefficient of the scale was 0.88, and the Cronbach coefficient of each dimension was 0.85, 0.60, and 0.66. Confirmatory factor analysis showed that the scale had reliable structural validity and was suitable for Chinese cultural background (18).

#### The Social Support Rating Scale

Social support was measured by the Social Support Rating Scale (SSRS), which was edited to Chinese version in 1999 by SY Xiao. It is a self-rating scale which include 10-item survey originally designed to assess three different dimensions of social support: subjective support (4 items), objective support (3 items), and support-seeking behavior (3 items). Subjective support reflects the perceived interpersonal supports which an individual can count on. Objective support reflects the degree of actual support an individual received in the past. Support-seeking behavior refers to the different patterns of behavior that an individual would utilize when trying to seek social support. A higher score indicates more social support. participants were all asked to use “yes” or “no” to indicate how they had felt in last 2 recent weeks. The Chinese version of SSRS shows a high reliability which exceeded 0.92 (19). The SSRS has been widely applied in Chinese population because of its high reliability and validity (20).

### Statistical Analysis

(1) The patients' demographic and disease characteristics were analyzed with descriptive statistics. (2) Pearson's correlation analysis was used to study the correlation between two continuous data, including TB-related stigma, social support, duration of disease, self-esteem, anxiety and depression. (3) Independent sample *T*-test or Mann-Whitney *U-*test as appropriate was used for comparison psychological health level (anxiety and depression)between TB patients with sensitive and MDR-TB. (4) Multiple linear stepwise regression analysis was conducted to explore risk factors for HADS anxiety and HADS depression, statistical test level ≤ 0.05. All statistical analyses were performed using Statistical Package for Social Sciences (SPSS) version 25.0 for Windows.

### Ethical Considerations

Ethical approval (LW-01) was obtained from the Institutional Ethical Review Board of Beijing Chest Hospital, Beijing, China. In addition, research permission was obtained from the hospital. All patients provided written informed consent to participate in this study. After data collection and analysis, we contacted the patients with depressive and anxiety symptoms by using wechat and suggested them undergo further evaluation at the department of mental disease.

## Results

### Demographic and Disease Characteristics

As shown in Table 1, among the 167 patients, 92 were male (55.09%) and 75 were female (44.91%). The age distribution was normal, and the proportion of patients with bachelor degree or less was higher, which is 67.66%. There were 65 (38.92%) patients with drug sensitive PTB and 102 (61.08%) patients with MDR-PTB. 100 patients were in the first treatment (59.88%) and 120 patients under treatment (71.86%).

**Table 1:** Characteristics of TB patients.

**Characteristic**	**Mean ± SD**	***n***	**(%)**
**Age**			
<18		2	1.20
18–25		35	20.96
26–30		32	19.16
31–40		38	22.75
41–50		39	23.35
51–60		17	10.18
Above 60		4	2.40
**Sex**			
Male		92	55.09
Female		75	44.91
**Education level**			
Beneath the bachelor		113	67.66
Bachelor		45	26.95
Master or above degree		9	5.39
Duration of Tuberculosis (month)	21.37 ± 28.44		
**Drug resistance**			
No drug resistance		65	38.92
Drug resistance		102	61.08
**Treatment times**			
Once		100	59.88
Twice		43	25.75
Third		8	4.79
Fourth		5	2.99
More		11	6.59
**Treatment outcome**			
Being cured		47	28.14
In the treatment		120	71.86

### Prevalence of Depressive and Anxiety Symptoms Among TB Patients

According to the HADS, the mean anxiety score of all patients was 5.96 (SD = 4.11), and depression score of all patients was 5.56 (SD = 4.58). A total of 49 (29.34%) TB patients were found to have anxiety symptoms and 50 (29.94%) TB patients were found to have depressive symptoms. A total of 40 (23.95%) TB patients had both depressive and anxiety symptoms. According to the GHQ-20, the mean anxiety score and mean depression score of all patients were 1.59 (SD = 1.91) and1.19 (SD = 1.72) respectively, and mean self-esteem score of all patients was 5.74 (SD = 2.77).

### Correlation Analysis of Stigma, Social Support, Self-Esteem, Duration of Disease, and Mental Health Scores of TB Patients

Table 2 shows that the psychological health problem is widely positively correlated with TSS and duration of disease (*p* < 0.01), but widely negatively correlated with social support and self-esteem (*p* < 0.01). In addition, for the scores of anxiety and depression, GHQ and HADS scale are highly correlated, indicating the consistency of the two scales and a strong validation of anxiety and depression scores.

**Table 2:** Correlation coefficient and *P-*value between two continuous data.

	**HADS anxiety**	**HADS depression**	**GHQ- self-esteem**	**GHQ-depression**	**GHQ-anxiety**	**TSS**	**Total score of social support**	**Objective support factor**	**Subjective supportive factor**	**Support-seeking behavior**	**Duration of disease**
HADS anxiety	1.000										
HADS depression	0.814^**^ (0.000)	1.000									
GHQ- self-esteem	–.0.622^**^ (0.000)	−0.655^**^ (0.000)	1.000								
GHQ-depression	0.598^**^ (0.000)	0.634^**^ (0.000)	−0.655^**^ (0.000)	1.000							
GHQ-anxiety	0.713^**^ (0.000)	0.691^**^ (0.000)	–.0.712^**^ (0.000)	0.656^**^ (0.000)	1.000						
TSS	0.595^**^ (0.000)	0.601^**^ (0.000)	−0.545^**^ (0.000)	0.485^**^ (0.000)	0.503^**^ (0.000)	1.000					
Total score of Social Support	−0.330^**^ (0.000)	−0.460^**^ (0.000)	0.382^**^ (0.000)	−0.416^**^ (0.000)	−0.329^**^ (0.000)	−0.414^**^ (0.000)	1.000				
Objective support factor	−0.248^**^ (0.001)	−0.400^**^ (0.000)	0.241^**^ (0.002)	−0.222^**^ (0.004)	−0.196^*^ (0.011)	−0.377^**^ (0.000)	0.619^**^ (0.000)	1.000			
Subjective supportive factor	−0.213^**^ (0.006)	−0.274^**^ (0.000)	0.308^**^ (0.000)	−0.314^**^ (0.000)	−0.220^**^ (0.004)	−0.235^**^ (0.002)	0.827^**^ (0.000)	0.166^*^ (0.032)	1.000		
Support-seeking behavior	−0.275^**^ (0.000)	−0.362^**^ (0.000)	0.242^**^ (0.002)	−0.369^**^ (0.000)	−0.329^**^ (0.000)	−0.328^**^ (0.000)	0.602^**^ (0.000)	0.305^**^ (0.000)	0.260^**^ (0.001)	1	
Duration of disease	0.448^**^ (0.000)	0.460^**^ (0.000)	−0.388^**^ (0.000)	0.452^**^ (0.000)	0.356^**^ (0.000)	0.287^**^ (0.000)	−0.244^**^ (0.001)	−0.156^*^ (0.044)	−0.148 (0.056)	−0.263^**^ (0.001)	1

** *p < 0.01*.

* *p < 0.05*.

*The number at the top of the box shows the correlation coefficient, and the number at the bottom of the box shows P-value*.

### Comparative Analysis of TB Patients With and Without Drug Resistance

Two mental health screening scales (HADS and GHQ) demonstrated that MDR-PTB patients were more likely to suffer from probable anxiety and depression than drug sensitive PTB patients without drug resistance. The median anxiety and depression score for the drug resistance patients was 7.0 and 6.5, for the without drug resistance patients was 3.0 and 2.0, respectively. Because of the heterogeneous of the variance, Mann-Whitney U test was conducted for comparison mental health scores between with drug resistance and without drug resistance groups. MDR-PTB Patients had significantly higher anxiety, depression, self-esteem and stigma scores than patients without drug resistance (*p* < 0.01), and there was no significant difference in total social support scores between the two groups.

### Factors Associated With Anxiety and Depression Among Patients With TB

Multiple linear stepwise regression analysis revealed that self-esteem, drug resistance, perceived TB stigma and duration of TB were statistically significant with anxiety. Self-esteem, social supports, perceived TB stigma and duration of TB were statistically significant with depression (Tables 3, 4).

**Table 3:** Stepwise multiple linear regression analysis results of anxiety.

**Model**	**Variance**	**Unstandardized coefficients**		**Standardized coefficients**	***R*^2^**	**Adjusted *R*^2^**
		***B***	**SE**	**β**		
1	(Constant)	11.238	0.650		0.340	0.335
	Self-esteem	−0.876	0.108	−0.583		
2	(Constant)	8.038	0.930		0.432	0.423
	Self-esteem	−0.748	0.104	−0.498		
	Drug resistance	3.203	0.706	0.315		
3	(Constant)	5.391	1.227		0.474	0.461
	Self-esteem	−0.584	0.113	−0.388		
	Drug resistance	2.496	0.718	0.246		
	TB stigma	0.224	0.071	0.251		
4	(Constant)	4.634	1.231		0.502	0.486
	Self-esteem	−0.497	0.115	−0.331		
	Drug resistance	2.058	0.720	0.202		
	TB stigma	0.224	0.069	0.251		
	Duration of tuberculosis	0.025	0.009	0.187		

**Table 4:** Stepwise multiple linear regression results of depression.

**Model**	**Variance**	**Unstandardized coefficients**		**Standardized coefficients**	***R*^2^**	**Adjusted *R*^2^**
		***B***	**SE**	**β**		
1	(Constant)	11.938	0.696		0.385	0.380
	Self-esteem	−1.033	0.115	−0.620		
2	(Constant)	18.219	1.481		0.476	0.468
	Self-esteem	−0.838	0.115	−0.503		
	Social support	−0.219	0.046	−0.324		
3	(Constant)	13.245	2.191		0.511	0.500
	Self-esteem	−0.686	0.122	−0.412		
	Social support	−0.166	0.048	−0.246		
	TB stigma	0.231	0.077	0.234		
4	(Constant)	11.592	2.218		0.539	0.525
	Self-esteem	−0.593	0.124	−0.356		
	Social support	−0.151	0.047	−0.223		
	TB stigma	0.226	0.075	0.228		
	Duration of tuberculosis	0.027	0.010	0.182		

## Discussion

The inextricable relationship between TB and mental disorders is well-known although less well-researched. Mental illnesses such as anxiety, depression may have a high comorbidity rate among people suffering from TB (21). However, there is a lack of research on the prevalence of anxiety and depression in Chinese patients with PTB, especially in patients with MDR-TB. We conducted a structured questionnaire in 167 hospitalized TB patients to assess prevalence of depression/anxiety symptoms, TB-related stigma and social support in patients, and to explore the impact of other factors on psychological health problems.

The prevalence of probable anxiety and depression in this TB population was 29.34 and 29.94% according to the HADS. Other studies determined that the prevalence of anxiety among patients with TB ranged from 18.37% in the China to 47.2% among hospitalized patients in Pakistan (3, 4), the prevalence of depression among patients with TB ranged from 17.73 to 48% in China (4, 7). This result is consistent with the findings of the prevalence of anxiety and depression noted among patients enrolled in previous studies, which suggests there is a higher prevalence of mental illness among TB patients. Our study also noted a strong positively correlation between the anxiety and depression scores of HADS and GHQ-20, which indicates our outcome of anxiety and depression have good reliability and validity.

Compared to TB patients without drug resistance, our study indicated that MDR-PTB patients were more likely to suffer from anxiety, depression, TB-related stigma and low self- affirmation. This is the first report to compare the psychological health problem, TB-stigma and social support between TB patients with and without drug resistance in China. This is consistent with a previous research in Serbia, which showed that MDR-TB cases are almost three times more frequently experienced subjective feeling of nervousness, sadness, used sedatives and stigma associated with TB compared to drug susceptible respondents (22). TB is stigmatized because of its associations with HIV, poverty, low social class, malnutrition, or disreputable behavior (10). MDR-TB patients are particularly affected having in mind long (20 month) and arduous treatment, regime consisted of powerful anti-TB drugs with severe side-effects that can affect mental health, higher case fatality and lower cure rate (23). Furthermore, the high score of psychological health problem noted among drug resistance TB patients may be associated with higher TB-related stigma and longer duration of treatment, which is more common in patients with drug resistance than patients without drug resistance. This could be concluded that drug resistance, TB-stigma and self-esteem have an influence on psychological health among TB patients.

One of the factors significantly associated with anxiety and depression was self-esteem. A previous study with hospitalized patients with TB, which showed that self-esteem scores dropped in accordance with category of depression, revealing that low self-esteem is a characteristic of depression (24). Besides, a study in Indonesia found a significant correlation between self-esteem with the incidence of anxiety in patients with MDR-TB (25). Stigmatization, negative emotions, social rejection, and isolation were reported by TB patients and could contribute to low self-esteem and impairment of psychosocial well-being (26). The reason of MDR-PTB patients have lower self-esteem might be that they had experienced more lacks of control over their self-care activities, more financial stress due to the longer duration of treatment, more restrictions on recreational and social activities, loss of independence, early retirement, role alterations and disruption in family life, then lead to the altered self-image and diminished self-esteem (27).

It's worth noting that poor social support was only significantly associated with depression and multi-drug resistance was only significantly associated with anxiety. This could indicate that lack of (poor) social support may lead to increased depression symptom and multi-drug resistance may more likely associated with anxiety symptom. On the other hand, good social support is vital for those with good health in prevention of depression, and multi-drug resistance have a greater contribution to anxiety symptoms.

In this study we found low self-esteem, high TB-related stigma and long duration of TB are all risk factors for psychological problems. We also found multi-drug resistance is riskier especially for anxiety, lower social support is risker for depression. Besides, MDR-PTB patients were more likely to suffer from anxiety, depression, TB-related stigma and low self- affirmation than drug sensitive PTB patients, which suggests that the whole society, from family members, doctors to government, need to pay attention to the mental health of MDR-PTB patients. In addition, measures to reduce psychological distress among patients with TB should include a more comprehensive care approach which include psychological treatment, family support, social support, government unemployment support and economic compensation. Limitations of sample size in this study should be considered, the results in current study should be further verified in studies with larger sample size. Furthermore, whether related psychopharmacological interventions can improve the mental state of TB patients should be further researched.

## Data Availability Statement

The original contributions presented in the study are included in the article/supplementary material, further inquiries can be directed to the corresponding authors.

## Ethics Statement

The studies involving human participants were reviewed and approved by Institutional Ethical Review Board of Beijing Chest Hospital, Beijing, China. The patients/participants provided their written informed consent to participate in this study.

## Author Contributions

LZ and LG designed the study and drafted the manuscript. YZ performed the experiments. KL, SQ, and WY performed clinical examination. All authors contributed to the article and approved the submitted version.

## Funding

This study was supported by the grant from the Ministry of Science and Technology, People's Republic of China (2018ZX10302302001009); this study was also supported by Beijing Municipal Administration of Hospitals Clinical Medicine Development of Special Funding Support (ZYLX201824) and Beijing Municipal Administration of Hospitals' Ascent Plan (DFL20181602). This work was also supported by CAS Key Laboratory of Mental Health, Institute of Psychology, Chinese Academy of Sciences.

## Conflict of Interest

The authors declare that the research was conducted in the absence of any commercial or financial relationships that could be construed as a potential conflict of interest.

## Publisher's Note

All claims expressed in this article are solely those of the authors and do not necessarily represent those of their affiliated organizations, or those of the publisher, the editors and the reviewers. Any product that may be evaluated in this article, or claim that may be made by its manufacturer, is not guaranteed or endorsed by the publisher.
